# Hyperexpression of tumor necrosis factor receptor 2 inhibits differentiation of myeloid‐derived suppressor cells by instigating apolarity during ageing

**DOI:** 10.1002/mco2.605

**Published:** 2024-06-12

**Authors:** Ming Wang, Yijie Han, Xiaohan Yao, Xixi Duan, Jiajia Wan, Xiaohan Lou, Yan Yan, Peiguo Zheng, Fazhan Wang, Linyu Zhu, Chen Ni, Zhenzhen Pan, Zihao Wang, Lin Chen, Zhaoqing Wang, Zhihai Qin

**Affiliations:** ^1^ Medical Research Center, The First Affiliated Hospital of Zhengzhou University Zhengzhou University Zhengzhou Henan China; ^2^ Key Laboratory of Protein and Peptide Pharmaceuticals, Institute of Biophysics Chinese Academy of Sciences Beijing China; ^3^ Clinical Laboratory the First Affiliated Hospital of Zhengzhou University Zhengzhou Henan China

**Keywords:** ageing, differentiation, MDSC, TNFR2

## Abstract

During the ageing process, TNF‐α can promote the expansion of myeloid‐derived suppressor cells (MDSCs). However, it remains unclear which receptor(s) of TNF‐α are involved in and how they modulate this process. Here, we report that TNFR2 hyperexpression induced by either TNF‐α or IL‐6, two proinflammatory factors of senescence‐associated secretory phenotype (SASP), causes cellular apolarity and differentiation inhibition in aged MDSCs. Ex vivo overexpression of TNFR2 in young MDSCs inhibited their polarity and differentiation, whereas in vivo depletion of *Tnfr2* in aged MDSCs promotes their differentiation. Consequently, the age‐dependent increase of TNFR2 versus unaltered TNFR1 expression in aged MDSCs significantly shifts the balance of TNF‐α signaling toward the TNFR2–JNK axis, which accounts for JNK‐induced impairment of cell polarity and differentiation failure of aged MDSCs. Consistently, inhibiting JNK attenuates apolarity and partially restores the differentiation capacity of aged MDSCs, suggesting that upregulated TNFR2/JNK signaling is a key factor limiting MDSC differentiation during organismal ageing. Therefore, abnormal hyperexpression of TNFR2 represents a general mechanism by which extrinsic SASP signals disrupt intrinsic cell polarity behavior, thereby arresting mature differentiation of MDSCs with ageing, suggesting that TNFR2 could be a potential therapeutic target for intervention of ageing through rejuvenation of aged MDSCs.

## Graphical abstract

Ageing in stem or progenitor cells often results in cellular apolarity, which impedes their mature differentiation. However, the induction of apolarity in aged myeloid‐derived suppressor cells (MDSCs) remains unexplored. This study reveals that the TNFR2 hyperexpression, triggered by either TNF‐α or IL‐6—two factors associated with the senescence‐associated secretory phenotype (SASP), causes JNK‐induced apolarity and inhibits differentiation in aged MDSCs.

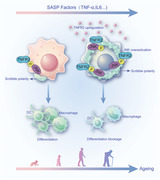

## 1 INTRODUCTION

Myeloid‐derived suppressor cells (MDSCs) are a heterogeneous group of cells that consists of myeloid progenitor cells and immature myeloid cells.^1^, ^2^ Under physical conditions, MDSCs can differentiate into mature granulocytes, dendritic cells (DCs), and macrophages.^1^, ^2^ Conversely, under various pathological conditions ranging from cancer to ageing, MDSCs fail undergo efficient differentiation. This results in their excessive and prolonged expansion and accumulation.^3^ Given their potent immunosuppressive capability, MDSCs form a significant component of the immunosuppressive microenvironment.^4^ Therefore, deepening understanding of the mechanisms that prevent MDSCs from maturing is crucial for mitigating their immunosuppressive effects.

With chronological age increases, MDSCs’ expansion and activation (enhancement of immunosuppressive capability) also progressively augment.^3^, ^5^, ^6^ The transcription factor NF‐κΒ is proved to play a central role in the generation of senescence‐associated secretory phenotype (SASP), especially TNF‐α and IL‐6.^7^, ^8^ Gradually increased proinflammatory cytokines of TNF‐α and IL‐6 serve as major extrinsic mediators for MDSC expansion with ageing.^3^, ^9^, ^10^ However, the key intrinsic pathways, especially the TNF‐α receptors, regulating MDSCs’ differentiation during ageing are elusive.

In vertebrates, TNF‐α exerts functions through its two receptors, TNFR1 and TNFR2.^11^ These receptors are extremely distinct in structure, expression, distribution, and signaling.^12^, ^13^ TNFR1, but not TNFR2, contains a death domain that triggers cell death, whereas TNFR2 dictates cell survival.^14^ TNFR1 is constantly expressed and ubiquitously exists on all types of cells; whereas TNFR2 is inducibly expressed and restrictedly appears in specific cell lines, such as immune, neuronal, and endothelial cells.^15^ Although both receptors can activate their shared default downstream pathway, NF‐kB signaling; the activation of the Jun N‐terminal kinase (JNK) pathway is dependent on TNFR2. This is because only TNFR2 possesses the TRAF2‐interacting‐motif necessary for JNK activation, unlike TNFR1.^14^ Therefore, TNFR2 expression state is a crucial determinant in maintaining the balance of TNFR1–TNFR2 signaling and in determining cellular fates, such as life or death.

Cell polarity is a fundamental cellular behavior required for diverse processes across all cell types, including asymmetric cell division in stem or progenitor cells. For instance, hematopoietic stem and progenitor cells (HSPCs) rely on asymmetric cell division to maintain their dual capabilities of self‐renewal into a daughter stem cell and differentiation into multiple cell lineages.^16^, ^17^ Accordingly, apolarity has been recognized as an intrinsic mechanism leading to differentiation blockage in aged HSPCs.^18^, ^19^ Although differentiation blockage in MDSCs has been observed in distinct pathological conditions,^20^ it remains unknown whether such developmental failure is attributable to cell polarity abnormality under such conditions, especially ageing process.

In *Drosophila*, Grindelwald is the homologous protein to vertebrate TNFR2 and overexpression of its intracellular domains is sufficient to induce sustained JNK activation.^21^ Interestingly, fly TNFR2 colocalizes to the apical zone with the apical cell polarity determinant Crumbs, coordinating Crumbs‐induced apolarity with JNK activation in epithelial tumors.^21^ Beside its role in integration of TNFR2 signaling, JNK is also a well‐known effector downstream from the noncanonical Wnt/planar cell polarity (PCP) pathway.^22^, ^23^ The TNF‐α/NF‐kB pathway plays a significant role in the expansion of MDSCs with ageing.^3^, ^9^ However, the specific receptor of TNF‐α implicated in this process remains elusive. Furthermore, the critical involvement of vertebrate TNFR2 in regulating cell polarity through JNK has yet to be investigated.

Our previous investigation has revealed that the TNF‐α/TNFR2 axis promotes expansion of tumor‐infiltrated MDSCs through inhibiting their apoptosis.^24^ Considering the established causal relationship between apolarity and differentiation failure in aged HSPCs, the critical role of fly TNFR2 in epithelial cell polarity, and the context‐dependent nature of MDSCs’ expansion, we hypothesized that TNFR2 could contribute to the expansion of ageing‐related MDSCs. The present study investigated whether TNFR2 disrupts the cell polarity of aged MDSCs, thereby hindering their mature differentiation progression and subsequent expansion during ageing.

## 2 RESULTS

### 2.1 TNFR2 hyperexpression in MDSCs with ageing

As increases of MDSCs’ expansion and activation are dependent on chronological age,^3^, ^5^, ^6^ we established a murine ageing model, in which the age of young and old wild‐type (WT) mice was 2−3 and 18−24 months, respectively. We found that aged MDSCs expressed increased levels of p16^Ink4a^ and p21^CIP^, two well‐known senescence markers and cell cycle inhibitors, compared with young MDSCs (Figures S1A–C). These data demonstrate the feasibility of the murine model of ageing.

**FIGURE 1:**
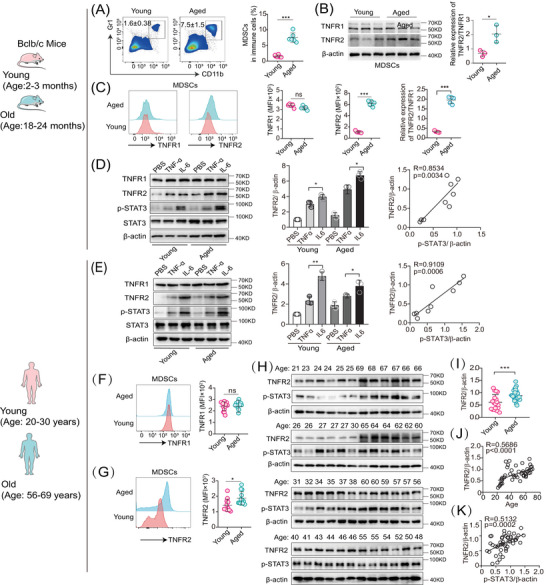
TNFR2 hyperexpression in aged MDSCs. (A) Representative flow cytometry analyses comparing the percentage of splenic Gr1^+^CD11b^+^ MDSCs between young and old mice (*n* = 6). (B) Immunoblotting detecting the expression of both TNFR1 and TNFR2 in splenic MDSCs from young and old mice. The ratio of TNFR2/TNFR1 was calculated using band density after quantification with β‐actin (*n* = 3). (C) Flow cytometric analyses of the expression of both TNFR1 and TNFR2 in splenic MDSCs from young and old mice (*n* = 5). (D and E) The expression of TNFR1 and TNFR2, along with phosphorylation and expression of STAT‐3, were examined in young and aged MDSCs from either murine spleen in (D) or human blood (E) (*n* = 3) following treatment with TNF‐α (10 ng/mL) or IL6 (5 ng/mL) for 30 min. In the middle panels, the relative TNFR2/β‐actin expression was determined by evaluating the band densities of TNFR2 over β‐actin (loading control). In the right panels, Pearson analysis of the correlation between TNFR2/β‐actin ratio and p‐STAT3/β‐actin. (F and G) Flow cytometric analysis of the expression of both TNFR1 and TNFR2 in blood MDSCs from healthy young and old human (*n* = 15). (H) TNFR2 expression and STAT‐3 phosphorylation in young and aged blood MDSCs from healthy young (*n* = 18) and old humans (*n* = 30). (I) The relative TNFR2/β‐actin expression was determined by evaluating the band densities of TNFR2 over β‐actin (loading control). (J and K) Pearson comparison analysis of the correlation between either TNFR2/β‐actin ratio (J, *n* = 48) or p‐STAT3/β‐actin (K) and human age. The results were reported as Spearman's correlation coefficient (R) and the corresponding *p*‐value. Data in panels (A, C, F, and G) are representative of at least three independent experiments and are presented as mean ± SD. Statistical analyses were performed using a *t*‐test. *p < 0.05, **p < 0.01, ***p < 0.001, and ns, not significant. MFI, mean fluorescence intensity.

We then determined whether MDSCs accumulate with ageing. Indeed, the fraction of splenic CD11b^+^ Gr‐1^+^ (a marker comprising Ly6G/Ly6C) MDSCs in old mice was significantly increased compared with those in young mice (Figure 1A). Such MDSCs’ age‐dependent accumulation coexisted in its two subpopulations, monocytic (CD11b^+^ Ly6G^−^ Ly6C^hi^) and granulocytic (CD11b^+^ Ly6G^+^ Ly6C^lo^) MDSCs (Figure S1D). In this study, both subpopulations of monocytic and granulocytic MDSCs (CD11b^+^ Gr‐1^+^) were analyzed consistently in all further experiments. These results confirmed the previous findings that MDSCs’ numbers are progressively increased with ageing.

We then checked whether ageing affects MDSCs’ activation. First, we compared the in vitro inhibitory effects of young and aged MDSCs on T cell proliferation through coculturing MDSCs with naive CD3^+^ T (CD4^+^ and CD8^+^) cells in the presence of concanavalin A (ConA). The results showed that the frequency of T cells cocultured with aged MDSCs was significantly decreased than those cocultured with young MDSCs (Figure S1E). Second, we compared the in vivo inhibitory effects of young and aged MDSCs on T cell proliferation by assessing ζ chain expression of T cells in young and old mice based on the observations that MDSCs inhibit T and NK T cell functions through downregulation of the ζ chain expression in their T cell receptor.^20^, ^25^ The results showed that old mice had a significantly reduced ζ chain expression in CD3^+^ T (CD4^+^ and CD8^+^) cells compared with young mice (Figure S1F). These results thus proved that the activation of aged MDSCs also increased with ageing.

The upregulation of TNFR2 in cancerous cells is a prevalent phenomenon.^26^ To explore whether this trend was also existed in aged MDSCs, we conducted a comparative analysis of TNFR2 expression between young and aged murine MDSCs using immunoblotting and flow cytometry. Our findings revealed that in aged MDSCs, the TNFR2 expression was significantly increased by about 300% (*p* < 0.05), whereas the TNFR1 expression remained unchanged, resulting in the TNFR2/TNFR1 ratio in aged MDSCs was double that in young MDSCs (Figures 1B and C).

The transcriptional factor STAT3 not only mediates MDSCs expansion but also induces TNFR2 upregulation responding to TNF‐α and IL‐6.^1^, ^26^ However, whether TNFR2 expression is inducible in MDSCs is unexplored. Given that TNF‐α and IL‐6, two key proinflammatory factors of SASP, are prevalent in the age‐related chronic inflammation microenvironment, we subjected both young (2–3‐month‐old) and aged (18–24‐month‐old) murine MDSCs to treatment with these two SASP factors. Immunoblotting results indicated that both TNF‐α and IL‐6 can induce TNFR2 expression in both young and aged murine MDSCs and the inducible effects of IL‐6 are stronger than those of TNF‐α, but they had no effects on TNFR1 expression (Figure 1D, left and middle panels). TNFR2 expression was significantly correlated with the degree of STAT3 phosphorylation in MDSCs (Figure 1D, right panel)

Next, we wondered whether the same phenomenon also existed in human MDSCs (CD45^+^ HLA‐DR^−^ CD33^+^ CD11b^+^). To this end, we isolated MDSCs from peripheral blood of healthy adults aged 20−82 years. According to the age correlation between mice and humans,^27^ we set up young (20–30‐year‐old) and old (56–69‐year‐old) age groups analogous to mice ages of young and old groups (Figure S1G). Similarly, we also observed that in human MDSCs, TNFR2, but not TNFR1, can be induced by TNF‐α and IL‐6 (Figure 1E, left and middle panels). TNFR2 expression was significantly correlated with the degree of STAT3 phosphorylation in MDSCs (Figure 1D, right panel). In human MDSCs, the upregulated expression of TNFR2 versus the stable expression of TNFR1 with ageing were also confirmed by flow cytometry (Figures 1F and G). Next, we examined the correlation between TNFR2 expression in human MDSCs with ages by immunoblotting. We found that human TNFR2 expression as well as STAT3 activation in aged MDSCs were significantly elevated compared with young MDSCs (Figures 1H, I, and S1H). Pearson's correlation coefficient analysis indicated that TNFR2 expression in MDSCs increased markedly with either age (Figure 1J) or the degree of STAT3 phosphorylation (Figure 1K). Taken together, these results indicate that intrinsic TNFR2 in MDSCs is inducible and its expression levels are escalated with increases in chronological age.

### 2.2 *Tnfr2* deficiency suppresses MDSC accumulation with ageing

**FIGURE 2:**
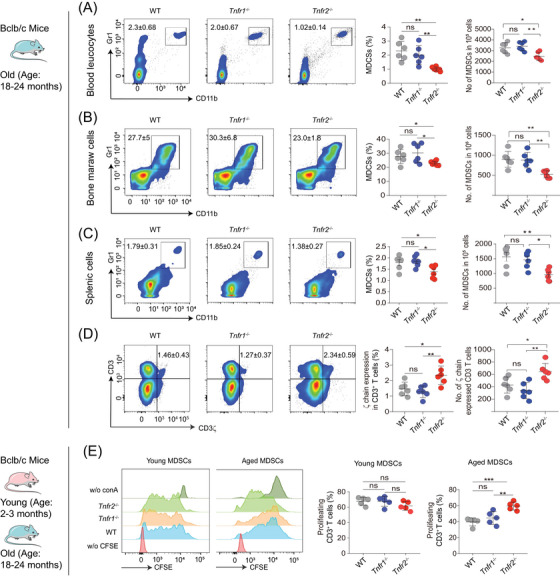
*Tnfr2* deficiency attenuates expansion of aged MDSCs. (A–C) Flow cytometric analyses of the proportion and number of Gr1^+^CD11b^+^ MDSCs in blood (A) bone marrow (B) and spleen (C) of old WT, *Tnfr1* ^−/−^ and *Tnfr2* ^−/−^ mice, respectively (*n* = 6). (D) Flow cytometry analyses of the percentage and number of CD3^+^CD3ζ^+^ T cells in old WT, *Tnfr1* ^−/−^, and *Tnfr2* ^−/−^ mice (*n* = 6). (E) CFSE‐labeled splenic T cells from young WT mice were cocultured with either young or aged splenic MDSCs from WT, *Tnfr1* ^−/−^, and *Tnfr2* ^−/−^ mice (*n* = 5) for 3 days. The percentage of proliferated CD3^+^ T cells was measured by flow cytometry. Data are representative of at least three independent experiments and are presented as mean ± SD. Statistical analysis was performed using one‐way ANOVA. *p < 0.05, **p < 0.01, ***p < 0.001, and ns, not significant. WT, wild type; CFSE, 5(6)‐carboxyfluorescein diacetate N‐succinimidyl ester; w/o (without); ConA, concanavalin A.

To explore whether TNFR2 is involved in the regulation of MDSC accumulation during ageing, we isolated young and aged MDSCs from the blood, spleen and bone marrow (BM) of WT, *Tnfr1* ^−/−^ *and Tnfr2* ^−/−^ knockout mice. Flow cytometry analysis revealed that the proportion of aged MDSCs at all sites from *Tnfr2* ^−/−^ mice was significantly reduced compared with those from *Tnfr1* ^−/−^ and WT mice (Figures 2A–C). In addition, there is no significant difference on the proportion of MDSCs between old *Tnfr1* ^−/−^ and WT mice. In contrast, the proportion of young counterpart MDSCs was similar among all three different mouse strains (Figures S2A–C). These findings demonstrate that *Tnfr2* deficiency can significantly ameliorate the accumulation of MDSCs in old mice.

Next, we asked whether TNFR2 affects the activation of MDSCs. Initially, we compared the in vivo inhibitory effects of aged MDSCs from WT, *Tnfr1* ^−/−^, and *Tnfr2* ^−/−^ mice. The results showed that *Tnfr2* ^−/−^ mice had a significantly increased ζ chain expression in CD3^+^ T (CD4^+^ and CD8^+^) cells compared with WT and *Tnfr1* ^−/−^ mice, demonstrating that aged *Tnfr2* ^−/−^ MDSCs exhibited diminished inhibitory effects on T cells (Figure 2D). Subsequently, we assessed the in vitro inhibitory effects of both young and aged MDSCs from WT, *Tnfr1* ^−/−^, and *Tnfr2* ^−/−^ mice on T cell proliferation by coculturing T cells with MDSCs in the presence of ConA. Our results indicated that the T cells, cocultured with all three types of young MDSCs did not show any difference in proliferation. In contrast, T cells cocultured with aged *Tnfr2* ^−/−^ MDSCs showed a significant increase in proliferation compared with those cocultured with aged WT and *Tnfr1* ^−/−^ MDSCs (Figure 2E). In addition, there were no statistically significant differences in the immunosuppressive activities of aged MDSCs between *Tnfr1* ^−/−^ and WT mice (Figure 2E). These results demonstrated that *Tnfr2* deficiency also inhibits the aged MDSCs’ immunosuppressive activities, implying that TNFR2 expression is positively associated with the expansion and activation of aged MDSCs.

### 2.3 *Tnfr2* deficiency attenuates MDSCs’ proliferation arrest without affecting their apoptosis

**FIGURE 3:**
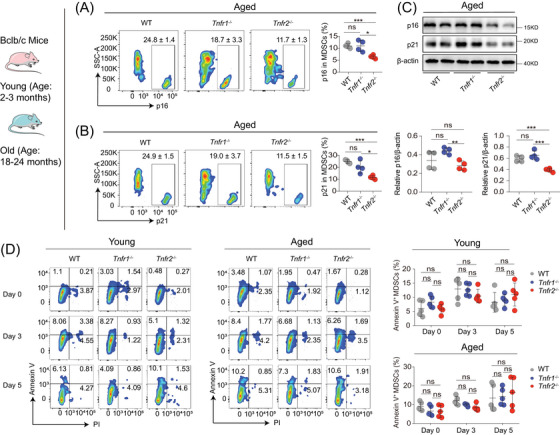
*Tnfr2* deficiency attenuates MDSCs’ cell cycle arrest without affecting their apoptosis. (A and B) Flow cytometry analysis of p16 and p21 expression in aged splenic MDSCs from WT, *Tnfr1* ^−/−^ and *Tnfr2* ^−/−^ mice (*n* = 4). (C) Immunoblotting detected the expression of p16 and p21 in splenic MDSCs from old mice (*n* = 2). (D) Splenic cells were isolated from young and old mice (*n* = 5) and the attached splenocytes were further cultured for indicated days. The percentage of apoptotic (Annexin V^+^) cells in Gr1^+^CD11b^+^ MDSCs at day 0, 3, and 5 were analyzed by flow cytometry (left panels). Bar graphs (right panels) show the percentage of Annexin V^+^ cells in Gr1^+^CD11b^+^ MDSCs. Data in panels (A, B, and D) are representative of at least three independent experiments and are presented as mean ± SD. Statistical analysis was performed using one‐way ANOVA. *p < 0.05, **p < 0.01, ***p < 0.001, and ns, not significant. WT, wild type; PI, propidium iodide.

Senescent cells are characterized by permanent cell cycle arrest and resistance to cell death,^28^, ^29^ which leads to their accumulation with ageing.^28^ To figure out how *Tnfr2* deficiency can cause MDSCs’ decrease with ageing, we first explored whether *Tnfr2* deficiency induces the cell cycle arrest in aged MDSCs. We chose to measure the expression of p16^Ink4a^ and p21^CIP^, two cell cycle inhibitors, because JNK is involved in transcriptional upregulation of both inhibitors in tumor cells.^30^, ^31^ Flow cytometry analysis showed that MDSCs from *Tnfr2* ^−/−^ mice expressed significantly lower levels of p16^Ink4a^ and p21^CIP^ than those from WT and *Tnfr1* ^−/−^ mice regardless of age (Figures 3A, B and S3A, S3B). Immunoblotting also showed that the protein levels of p16^Ink4a^ and p21^CIP^ in aged MDSCs from *Tnfr2* ^−/−^ mice were reduced compared with those from WT and *Tnfr1* ^−/−^ mice (Figure 3C). Consistently, mRNA‐sequencing (RNA‐seq) analysis on aged MDSCs isolated from the splenocytes of WT, *Tnfr1* ^−/−^, and *Tnfr2* ^−/−^ mice confirmed the enrichment of gene signatures associated with ageing in WT versus *Tnfr2* ^−/−^ (Figures S3C and S3D). Furthermore, the mRNA levels of some SASP elements of *Tnf‐α, Il6, Il1b*, and *Mcp1* were significantly decreased in *Tnfr2* ^−/−^ MDSCs compared with those in WT and *Tnfr1* ^−/−^ MDSCs (Figure S3E). These results demonstrated that *Tnfr2* deficiency attenuates MDSCs’ proliferation arrest, excluding the possibility that decreased accumulation of aged MDSCs related with *Tnfr2* deficiency is due to decreased proliferation.

Next, we wondered whether the MDSCs’ reduction in aged *Tnfr2* ^−/−^ mice was due to the reduced resistance of *Tnfr*2^−/−^ MDSCs to cell death. We thus determined apoptosis in young and aged MDSCs from WT, *Tnfr1* ^−/−^, and *Tnfr2* ^−/−^ mice by staining cells with anti‐annexin V antibody. The MDSCs were cultured in vitro for 3 or 5 days in the presence of granulocyte‐macrophage colony‐stimulating factor (GM‐CSF), and the cells isolated immediately were set as the 0‐day control group. Flow cytometric data showed that no different apoptosis was found in either young or aged MDSCs of all three 0‐, 3‐, and 5‐day groups from WT, *Tnfr1* ^−/−^, and *Tnfr2* ^−/−^ mice (Figure 3D), demonstrating that the decrease in proportion of MDSCs in *Tnfr2*‐deficient mice is not caused by an increase in apoptosis. Altogether, these results exclude the possibility that *Tnfr2* deficiency‐related reduction of aged MDSCs’ accumulation is attributable to a decrease in proliferation or an increase in cell death, implying that the reduction in MDSCs associated with *Tnfr2* deficiency could be a result of increased mature differentiation of aged MDSCs.

### 2.4 *Tnfr2* deficiency alleviates differentiation blockage in aged MDSCs

**FIGURE 4:**
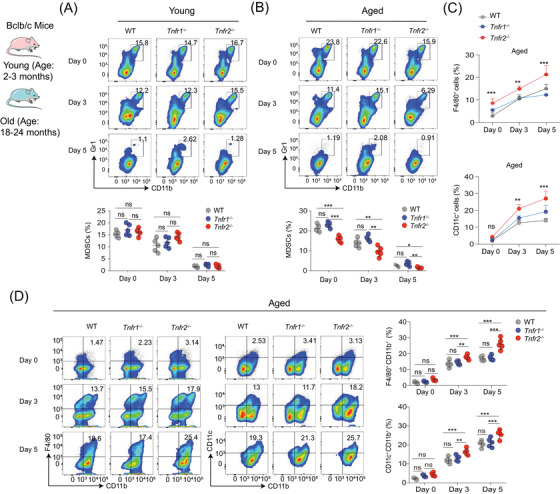
*Tnfr2* deficiency alleviates differentiation blockage in aged MDSC. Both young and aged splenic cells, isolated from WT, *Tnfr1* ^−/−^ and *Tnfr2* ^−/−^ mice, were further cultured for 3 or 5 days. Flow cytometry was used to detect the proportion of different cell lineages within these cell cultures. (A and B) The proportion of young and aged Gr1^+^CD11b^+^ MDSCs among CD45^+^ immune cells at days 0, 3, and 5. (C) The proportion of F4/80^+^ and CD11c^+^ cells in aged splenic immune cells at days 0, 3, and 5. (D) The proportion of F4/80^+^CD1b^+^ macrophages and CD11b^+^CD11c^+^ dendritic cells in aged splenic cells at days 0, 3, and 5. Data are representative of at least three independent experiments and are presented as mean ± SD. Statistical analysis was performed using one‐way ANOVA. *p < 0.05, **p < 0.01, ***p < 0.001, and ns, not significant. WT, wild type.

We next investigated whether *Tnfr2* deficiency impacts the mature differentiation of MDSCs with ageing. To this end, we isolated young and aged splenocytes from WT, *Tnfr1* ^−/−^, and *Tnfr2* ^−/−^ mice and cultured these cells for 3 or 5 days in the presence of GM‐CSF for their mature differentiation. The splenocytes immediately isolated without incubation with GM‐CSF were set as the 0‐day group. Analysis of cell phenotypes revealed that in both young and old mice, the percentages of MDSCs in the 3‐ and 5‐day groups were significantly reduced compared with the 0‐day group (Figures 4A and B). This suggests that either young or aged MDSCs retain their differentiation capacity. On days 0, 3, and 5, the percentages of young MDSCs were comparable among the WT, *Tnfr1* ^−/−^, and *Tnfr2* ^−/−^ mice (Figure 4A). In contrast, the percentages of aged *Tnfr2* ^−/−^ MDSCs were significantly lower than those of aged WT and *Tnfr1* ^−/−^ MDSCs on days 0, 3, and 5 (Figure 4B). These findings clearly demonstrate that *Tnfr2* deficiency only affects the differentiation of MDSCs from old, but not young mice.

Correspondingly, we observed that on days 3 and 5, the number and percentages of F4/80^+^ macrophages and CD11c^+^ DCs in aged *Tnfr2* ^−/−^ splenocytes cultures were significantly elevated compared with those in aged WT and *Tnfr1* ^−/−^ MDSCs cultures (Figures 4C and S4A–C). A phenotype analysis revealed that aged *Tnfr2* ^−/−^ splenocytes displayed a significantly higher differentiation capacity into macrophages and DCs than aged WT and *Tnfr1* ^−/−^ splenocytes (Figure 4D). On day 0, the percentages of macrophages and DCs were similar among the WT, *Tnfr1* ^−/−^, and *Tnfr2* ^−/−^ MDSCs. However, on days 3 and 5, old *Tnfr2* ^−/−^ splenocytes displayed a significantly greater differentiation capacity into macrophages and DCs as compared with aged WT and *Tnfr1* ^−/−^ MDSCs (Figure 4D). RNA‐seq analysis on three aged MDSCs revealed that gene signatures associated with high differential potential were enriched in old *Tnfr2* ^−/−^ compared with aged WT and *Tnfr1* ^−/−^ MDSCs (Figures 4D and E). In addition, *Tnfr2* knockdown in MSC2 cells also enhanced their mature differentiation (Figures S4F and G). These findings elucidated that *Tnfr2* deficiency can alleviate differentiation blockage of aged MDSCs, implying that TNFR2 is a potential target for reducing MDSCs’ accumulation with ageing by alleviating aged MDSCs’ differentiation blockage.

### 2.5 TNFR2 elevation in young MDSCs impairs cell polarity and differentiation

**FIGURE 5:**
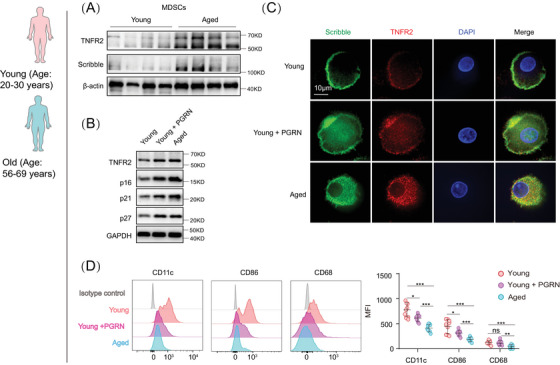
Hyperexpression of TNFR2 in young human MDSCs disrupts their cell polarity and arrests their differentiation. Human MDSCs, both young and aged, were isolated from healthy donors. The young MDSCs were treated with or without the TNFR2 agonist PGRN (200 ng/mL) for 24 h. (A) Immunoblots showing the expression levels of TNFR2 and Scribble in both young and aged MDSCs. (B) The expression of TNFR2, p16, p21, and p27 in aged MDSCs was compared with that in young MDSCs treated with or without PGRN. (C) Localization analysis of TNFR2 (red) and Scribble (green) by immunofluorescence staining and nuclei were stained with DAPI (blue). Scale bar: 10 µm. (D) The expressions of CD11c, CD86, and CD68 in MDSCs from both young and old human (*n* = 9) was evaluated by flow cytometry. Data are representative of at least three independent experiments and are presented as mean ± SD. Statistical analysis was performed using one‐way ANOVA. *p < 0.05, **p < 0.01, ***p < 0.001, and ns, not significant. PGRN, progranulin; MFI, mean fluorescence intensity.

We next wondered whether the differentiation blockage associated with TNFR2 hyperexpression is due to the disruption of cell polarity behaviors in aged MDSCs. RNA‐seq analysis of three different types of aged MDSCs revealed that *scribble* mRNA, encoding a polarity determinant, was higher in aged *Tnfr2* ^−/−^ compared with aged WT and *Tnfr1* ^−/−^ MDSCs (Figure S5A). Therefore, scribble was used as a polarity marker in this research. Immunoblotting results showed that Scribble is expressed in MDSCs and its expression level is also elevated in aged MDSCs compared with young MDSCs, similar to that of TNFR2 (Figure 5A).

To mimic TNFR2 hyperexpression in naturally aged MDSCs, we used progranulin (PGRN), an agonist of TNFR2,^32^ to treat young murine and human MDSCs. PGRN treatment in young MDSCs induced a significant increase of TNFR2 expression that was comparable to that observed in aged MDSCs (Figure 5B). Furthermore, PGRN treatment confirmed the positive correlation between TNFR2 hyperexpression and proliferation arrest because PGRN‐treated young MDSCs (both mice and human) expressed significantly higher levels of senescence markers, p16^Ink4a^, p21^CIP^, and p27^kip1^, compared with the control cells (Figures 5B and S5B).

Next, the aforementioned cells were costained with antibodies of TNFR2 and Scribble to examine TNFR2's localization and MDSCs’ polarity behaviors. In both mouse and human young MDSCs, TNFR2 was found to be colocalized with Scribble in the polarized zone. In contrast, in aged and PGRN‐treated young MDSCs, such polarized localizations of TNFR2 and Scribble disappeared; instead, diffused localizations of both proteins appeared (Figures 5C and S5D). These findings suggest that TNFR2 plays sufficient roles in MDSCs’ cellular polarity and that its hyperexpression can induce a loss of cell polarity.

To understand the roles of TNFR2 hyperexpression‐caused apolarity in MDSCs’ differentiation, we next examined the above cells’ phenotypes. The human and mouse MDSCs were incubated with GM‐CSF for 5 days and the young MDSCs were cotreated with or without PGRN for 24 h. Cell phenotype analysis showed that PGRN‐treated young human MDSCs exhibited a significantly lesser differentiation capacity into macrophages (CD86^+^) and DCs (CD11c^+^) as compared with young MDSCs without PGRN treatment (Figure 5D). The differentiation capacity of PGRN‐treated young murine MDSCs was decreased to the similar degree as such in the aged murine MDSCs (Figure S5D). Next, we monitored the behaviors of MDSCs’ polarity in response to TNF‐α or IL‐6 stimulation in both young and aged cells. We found that MDSCs’ polarity was reduced in both young and aged MDSCs (Figure S5E). Furthermore, we examined the behaviors of MDSCs’ polarity in response to STAT3 inhibitor or in combination with TNF‐α treatment in aged MDSCs. The results in Figures S5F–I demonstrated that inhibition of STAT3 can counteract the cell polarity loss and inhibition of MDSCs differentiation induced by TNF‐α. These results thus certified the sufficient roles of TNFR2 in regulating MDSCs’ differentiation, demonstrating that TNFR2 hyperexpression can perturbate aged MDSCs’ cell polarity to block their mature differentiation with ageing.

### 2.6 TNFR2 hyperexpression is associated with JNK overactivation in aged MDSCs

**FIGURE 6:**
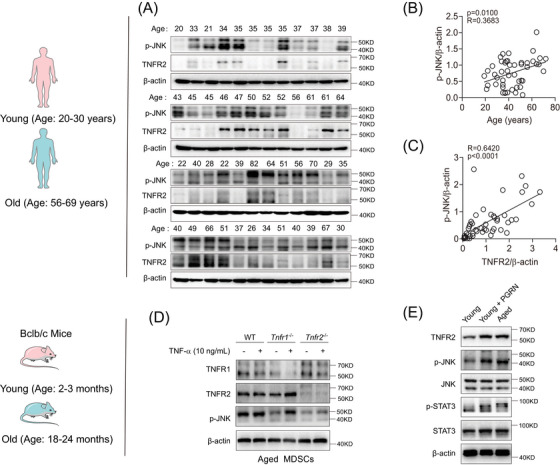
Hyperexpression of TNFR2 induces JNK overactivation in MDSCs. (A–C) Analysis of the JNK activation in human blood MDSCs from both healthy young and old individuals (*n* = 48). (A) Immunoblotting detected JNK phosphorylation and β‐actin expression in human MDSCs. (B and C) Pearson comparison analysis of the correlation between the p‐JNK/β‐actin ratio and either human age (B) or the TNFR2/β‐actin ratio (C). The resulting Spearman's correlation coefficient (R) and corresponding *p* value are reported. (D) The expression levels of TNFR1, TNFR2, and Scribble, and the phosphorylation of JNK in aged MDSCs isolated from spleens of WT, *Tnfr1* ^−/−^, and *Tnfr2* ^−/−^ mice in the presence or absence of TNF‐α (10 ng/mL). (E) The expression of TNFR2, JNK and STAT3, along with the phosphorylation of JNK and STAT3 in aged or young splenic MDSCs. The young cells were treated with or without PGRN (200 ng/mL) for 24 h. WT, wild type; PGRN, progranulin.

*Drosophila* TNFR2 participates in epithelial cell polarity in a JNK‐dependent manner,^21^ we next checked whether vertebrate TNFR2 also relies on JNK activation to regulate MDSCs’ cell polarity and differentiation. Using human MDSCs, we performed immunoblotting to assess JNK activation. We found that JNK activation was also increased in aged MDSCs compared with young MDSCs (Figures 6A and S6A). Pearson's correlation coefficient analysis revealed that JNK activation was also positively correlated with both human age and TNFR2 expression in MDSCs (Figures 6B and C).

Next, we explored the influence of TNFR2 expression on JNK activation in MDSCs. Immunoblotting results demonstrated that TNF‐α‐treated or untreated aged *Tnfr2* ^−/−^ MDSCs exhibited reduced JNK activities compared with WT and *Tnfr1* ^−/−^ MDSCs (Figure 6D), indicating that TNFR1 has little effect on JNK activation. RNA‐seq analysis verified the positive correlation between JNK activation and TNFR2 expression in aged MDSCs (Figures S6B and C). Such JNK activation patterns were also validated in MSC2 cells, in which the JNK activation stimulated with or without TNF‐α was impaired by *Tnfr2* knockdown (Figure S6D). Then, we treated young murine MDSCs with PGRN and found that, compared with untreated cells, PGRN‐treated young murine MDSCs showed significantly increased JNK activation, and their phosphorylation levels were comparable to those in naturally aged murine MDSCs (Figure 6E). Taken together, these results illustrated that TNFR2 hyperexpression induces biased overactivation of JNK in aged MDSCs.

### 2.7 JNK inhibition mitigates apolarity and differentiation blockage in aged MDSCs

**FIGURE 7:**
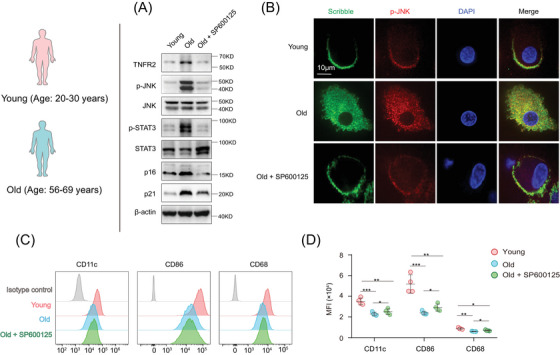
Inhibition of JNK activity in aged human MDSCs partially restores their cell polarity and differentiation. MDSCs from both young and aged humans were isolated from healthy donors and the aged MDSCs were treated with or without the JNK inhibitor SP600125 (40 nM) for 24 h. (A) Immunoblotting detected the expression of TNFR2, JNK, STAT3, p16, and p21, as well as the phosphorylation of JNK and STAT3 in MDSCs in the presence or absence of SP600125. (B) Immunofluorescence staining the localization of Scribble (green) and TNFR2 (red) in MDSCs in the presence or absence of SP600125. Nuclei (blue) were stained with DAPI. Scale bars: 10 µm. (C and D) Flow cytometry examination of the expression of CD11c, CD86, and CD68 in MDSCs isolated from young and old human (*n* = 9). MFI, mean fluorescence intensity. Data are representative of at least three independent experiments and are presented as mean ± SD. Statistical analysis was performed using one‐way ANOVA. *p < 0.05, **p < 0.01, and ***p < 0.001.

We next asked whether inhibition of JNK activity in aged MDSCs could mitigate their cell apolarity and differentiation blockage. JNK inhibitor, SP600125, was applied to aged murine and human MDSCs, while untreated young MDSCs were set as positive control. The suppression of JNK activity by SP600125 in aged human MDSCs was confirmed by the decreased levels of phosphorylated JNK and unchanged levels of JNK expression. Consistent with the previous report, JNK suppression reduced the STAT3 phosphorylation.^33^ In addition, the expressions of TNFR2, p16^Ink4a^, and p21^CIP^ were also reduced (Figures 7A and S7A).

Subsequently, the aforementioned cells were costained with antibodies targeting TNFR2 and Scribble to monitor alterations in cell polarity upon JNK inhibition. The immunostainings indicated that SP600125‐treatment in aged mice and human MDSCs partially mitigated their apolarity, and the polarized localizations of TNFR2 and Scribble re‐appeared following JNK inhibition (Figures 7B and S7B). These cells were then incubated with GM‐CSF for 5 days, and aged MDSCs were cotreated with or without SP600125 for a duration of 24 h. Cell phenotype analysis showed that SP600125‐treated aged MDSCs exhibited a significantly enhanced differentiation capacity into macrophages and DCs compared with aged MDSCs without SP600125 treatment (Figures 7C, D, and S7C). These results demonstrated that vertebrate TNFR2's influence on MDSCs’ polarity and differentiation also contingent upon the JNK cascade, suggesting that the hyperexpression of TNFR2 leading to JNK overactivation contributes to the apolarity and differentiation blockage observed in aged MDSCs with ageing.

## 3 DISCUSSIONS

A comprehensive understanding of MDSC expansion under pathological conditions needs to profoundly deciphering the mechanisms arresting MDSCs’ differentiation toward mature myeloid cell lineages. Here we reveal that SASP factors‐induced TNFR2 hyperexpression causes a loss of cell polarity and a blockage in mature differentiation in MDSCs with ageing, at the molecular, cellular, in vivo, and clinical levels. Thus, this study provides insights into the mechanisms by which proinflammatory SASP signals restrict the immature differentiation of MDSCs during organism ageing.

A key unsolved question in comprehending the mechanisms of MDSCs’ expansion is discovering intrinsic factors that interfere with their normal differentiation.^1^, ^2^ While it is well established that STAT3 is the chief transcription factor involved in the induction of MDSCs’ expansion,^1^, ^2^ its transcriptional effectors in preventing MDSCs’ differentiation remains unclear. In cancerous contexts, STAT3 controls TNFR2 hyperexpression responding to IL‐6, a member of the proinflammatory cytokine family that promotes MDSCs’ expansion.^26^ Our findings indicate that both TNFR2 expression and STAT3 activation were elevated simultaneously in MDSCs with ageing, suggesting that TNFR2 probably is a crucial intrinsic effector of the IL‐6/STAT3 pathway for MDSC expansion under pathological conditions, particularly in relation to ageing.

Under physiological conditions, the generation of MDSCs belongs to a normal process within myelopoiesis. This involves the differentiation of HPSCs into common myeloid progenitor cells, which subsequently develop into MDSCs for the generation of mature myeloid cells.^1^, ^2^ However, under pathological settings, especially chronic inflammation, various factors, such as IL‐6 and TNF‐α, prevent MDSCs’ differentiation and lead to their marked expansion.^1^, ^2^, ^20^ Inflammageing, characterized by its chronicity, can enable MDSCs to expand progressively with ageing.^34^ During ageing, senescent cells‐generated SASP contributes to inflammageing.^34^ We find that both chemokines can induce TNFR2 expression in MDSCs, suggesting that TNFR2 hyperexpression is the key link to creating a vicious cycle involving NF‐kB generating SASP and STAT3 driving MDSC expansion, two of which reinforce one another with ageing. However, our study still has some limitations in identifying alternative mechanisms underlying TNFR2 hyperexpression during the aging process.

Cell polarity‐dependent asymmetric division is a prerequisite for stem and progenitor cells (SPCs) to distribute fate determinants asymmetrically into daughter cells with different phenotypes.^35^, ^36^ Accordingly, apolarity plays causal roles in various differentiation defects in SPCs.^37^, ^38^ However, only limited research has explored the necessities of cell polarity in HSPCs’ differentiation, given that cell polarity is well understood in nonhematopoietic cells, especially in epithelia. Although JNK is an essential effector of the noncanonical Wnt/PCP pathway in epithelia, its role in HSPCs’ polarity is not fully clear. We found that inhibiting JNK can attenuate the apolarity of aged MDSCs and partially restore their differentiation capacity. This provides the first evidence that TNFR2/JNK is a pivotal signaling pathway controlling cell polarity and differentiation in MDSCs. Therefore, it is worth further investigation into the roles of TNFR2/JNK in differentiation of other hematopoietic or nonhematopoietic SPCs.

Our findings demonstrate a negative correlation between the expression levels of TNFR2 and mature differentiation in aged MDSCs. On the one hand, in vivo TNFR2 deficiency in aged MDSCs’ promotes their mature differentiation. On the other hand, ex vivo hyperexpression of TNFR2 in young MDSCs inhibits their mature differentiation. The sufficient roles of TNFR2 hyperexpression in preventing mature differentiation of aged MDSCs indicate that it is worth further exploring whether other possible mechanisms are also crucial for TNFR2 hyperexpression in MDSCs, such as epigenetic dysregulation.

It is clear that inevitable increase of senescent immune cells plays the major role in driving organismal ageing.^39^ Our research indicates a positive correlation between TNFR2 expression and the expression of senescence markers, specifically p16^Ink4a^ and p21^CIP^. Furthermore, we discovered that an elevation of TNFR2 expression in young MDSCs can precipitate their pre‐mature onset of senescence. Therefore, our findings suggest that TNFR2 probably is also a potential target for “rejuvenating” interventions of TNFR2^+^ immune cells, especially TNFR2^+^ HSPCs, with the aim of extending both health‐span and life‐span in humans.

To our knowledge, our findings provide the first evidence on how extrinsic proinflammatory SASP factors impair intrinsic cell polarity behaviors and differentiation programs of MDSCs during ageing. This will significantly contribute to a comprehensive understanding of the key molecular and cellular players that regulate the expansion of MDSCs with ageing. Therefore, this study is expected to shed light on the general mechanism by which SASP factors disrupt intrinsic cell polarity behaviors, leading to differentiation failure and senescence of MDSCs, or potentially other stem or progenitor cells, through the induction of TNFR2 hyperexpression during ageing.

## 4 MATERIALS AND METHODS

### 4.1 Mouse husbandry

BALB/c mice were purchased from Beijing Vital River Laboratory Animal Technology. *Tnfr1* ^−/−^ and *Tnfr2* ^−/−^ mice, on a BALB/c background, were generated by our laboratory as previously described.^40^ All mice were maintained on a chow diet and housed in specific pathogen‐free animal facilities. All animal testing and experimental research were performed using sex‐ and age‐matched mice, adhering to the guidelines set by the institutional Animal Care and Use Committee of First Affiliated Hospital of Zhengzhou University (2020‐KY‐249).

### 4.2 MDSCs isolation and culture

Mice were euthanized with 100% isoflurane, after which their BM, spleen, and blood were collected. For the isolation of splenic MDSCs, spleens were disaggregated into a 10 mL solution of RPMI‐1640 culture medium. BM and blood MDSCs were isolated from mouse femurs and submandibular vein, respectively. Thereafter, the cell suspension was filtered through 70 µm cell strainers, and the erythrocytes were lysed with red blood cell lysis buffer (R1010; Solarbio). Then, the cell suspension was washed with 1× phosphate‐buffered saline (PBS) and MDSCs were isolated using the MDSC isolation kit (#19867; STEM CELL), according to the manufacturer's instructions. The purity of the MDSCs was confirmed as CD45^+^Gr1^+^CD11b^+^ by flow cytometry. The freshly isolated MDSCs were cultured in RPMI‐1640 culture medium (Gibco), supplemented with 10% fetal bovine serum (PAN Biotech), 100 IU/mL penicillin, and 100 µg/mL streptomycin (Gibco), at 37°C in a 5% CO_2_ incubator. Additionally, the MDSC cell line MSC2 was cultured in RPMI‐1640 complete medium.

### 4.3 Human blood MDSCs collection

All human samples were obtained from healthy donors (*n* = 98; age, 44.88 ± 18.19 years; female: 47; male: 51) at the First Affiliated Hospital of ZhengZhou University. The study was conducted in accordance with informed consent guidelines from the patients and ethical standards set by the Ethics Committee of First Affiliated Hospital of Zhengzhou University (2020‐KY‐249). For the purification blood MDSCs, gradient Ficoll‐Hypaque (Sigma–Aldrich) and the EasySepTM HLA Chimerism Whole Blood CD33 Positive Selection Kit (Cat: 17885; STEMCELL Technologies) were used. The purification of human blood MDSCs was identified as CD45^+^HLA‐DR^−^CD33^+^CD11b^+^ by flow cytometry. These freshly isolated MDSCs were then cultured in RPMI‐1640 culture medium for further differentiation experiments or collected for immunoblotting and real‐time (RT)‐PCR analysis. Notably, healthy individuals did not have a history of cancer or clinical symptoms of diabetes at the time of enrollment.

### 4.4 Immunoblotting analysis

The primary MDSCs and MSC2 were lysed using radio‐immunoprecipitation assay buffer (K1423; Solarbio). Protein concentrations were determined using the Pierce BCA Protein Assay Kit (PC0020; Solarbio). A total of 20 µg protein was separated by sodium dodecyl sulfate‐polyacrylamide gel electrophoresis, followed by electrophoretically transferring to nitrocellulose filter membranes (HATF00010; Millipore). The membranes were then visualized through an electrochemiluminescence immunoblot detection system. All uncropped western blots are available in Figure S8.

### 4.5 Flow cytometry

Single‐cell suspensions, prepared from BM, spleen or blood, were lysed to remove the erythrocyte. Then, a total of 1 × 10^6^ single‐cell suspensions were incubated with an anti‐CD16/CD32 antibody at 4°C for 10 min. The subsequent procedures were carried out as outlined in our previous report.^41^ Cells were stained with fluorescent‐labeled mouse‐specific monoclonal antibodies. Then, the collected cells were blocked with a 3% bovine serum albumin (BSA) solution in PBS for 30 min. For staining intercellular proteins, the cells need to be fixed with intracellular fixation and permeabilization buffer (eBioscience) for 30 min, washed with permeabilization buffer (Invitrogen), and collected at 746*g* for 5 min at 4°C. Then cells were resuspended and incubated on ice with the corresponding flow cytometric antibodies or isotype IgG control antibody for 30 min at 4°C. After washing with permeabilization buffer and collection at 746*g* for 5 min at 4°C, the cells were analyzed using a FACSCalibur or FACSAria IIIu (BD Biosciences) flow cytometer. Flow cytometry data were analyzed using FlowJo V10 software (TreeStar).

### 4.6 MDSCs differentiation

MDSCs, or splenocytes, were cultured in the presence of GM‐CSF for indicated time interval or treated with either PGRN (200 ng/mL) or SP600125 (40 nM) for 24 h. Flow cytometry was used to detect the expression of F4/80, CD80, and CD11c in CD45^+^Gr1^+^CD11b^+^ mouse MDSCs, or CD68, CD86, and CD11c in CD45^+^HLD‐RA^−^CD33^+^CD11b^+^ human MDSCs. All experiments were conducted at least three times to ensure consistency and reliability.

### 4.7 RNA isolation and quantitative RT‐PCR

Total mRNA was isolated from human monocytes or mice splenic MDSCs with RNAiso Plus (9109; Takara, Tokyo, Japan), and complementary DNA (cDNA) was synthesized using the PrimeScript RT Master Mix (RR036A; Takara). cDNA was quantified by RT‐PCR using TB Green Premix Ex Taq II (RR820A; Takara) on an ABI PRISM 7300HT Sequence Detection 542 System (Applied Biosystems). The primers used are following. Mouse *mcp1*: forward, 5′‐GAA GGA ATG GGT CCA GAC AT; reward, 5′‐ACG GGT CAA CTT CAC ATT CA. Mouse *tnf‐a*: forward, 5′‐GAC GTG GAA CTG GCA GAA GAG; reward, 5′‐TTG GTG GTT TGT GAG TGT GAG. Mouse *il‐6* forward, 5′‐TAG TCC TTC CTA CCC CAA TTT CC; reward, 5′‐TTG GTC CTT AGC CAC TCC TTC. Mouse *il‐1b* forward, 5′‐TTC AGG CAG GCA GTA TCA CTC; reward, 5′‐GAA GGT CCA CGG GAA AGA CAC. Mouse *Tnfr1* forward, 5′‐GGC AGT GCA TAC CTG TTT TTG; reward, 5′‐AAA TAC CCC ACT CTC TGA CAG T. Mouse *Tnfr2* forward, 5′‐ACA CCC TAC AAA CCG GAA CC; reward, 5′‐AGC CTT CCT GTC ATA GTA TTC CT. Human *Tnfr1*, forward, 5′‐CTC AGT TCA TAC CGC TGG GG; reward, 5′‐GTG GGG ACT TGT GGT GAC AT. Human *Tnfr2* forward, 5′‐ACT TGC CTG CCG ATA AGG C; reward, 5′‐GCT CCC ACC TCT TAC CTG AG.

### 4.8 TNFR2 knockdown

MSC2 cells were transfected with either TNFR2 shRNA1 (5′‐TGA TGT TAG GAC TGG TGA), TNFR2 shRNA2 (5′‐TGA TGT TAG GAC TGG TGA A) or a negative control shRNA sequence (5′‐TTC TCC GAA CGT GTC ACG T) when the confluence reached 60%, as per the manufacturer's instructions (Genechem Co., LTD). The knockdown cells were selected with 10 µg/mL of neomycin. The knockdown efficiency was detected by immunoblotting.

### 4.9 Immunofluorescence

MDSCs, cultured in eight‐well plates (Sigma), were initially washed with PBS and subsequently fixed with 4% formaldehyde for 10 min. Following this, they were permeabilized with 0.1% Triton X‐100 for 10 min. Subsequently, the cells were incubated in a solution containing 5% BSA‐PBS for 1 h. The cells were then stained with either anti‐Scribble (Novus Biologicals), anti‐TNFR2 (Cell Signaling Technology), or anti‐p‐JNK antibody (Thermo) overnight at 4°C. This was followed by staining with an Alexa fluor 555‐labeled secondary antibody (Invitrogen) for 1 h. The nuclei were counter‐stained with 4′,6‐diamidino‐2‐phenylindole (DAPI) (0.5 µg/mL in PBS) for 5 min at room temperature. Fluorescence images were captured using a confocal laser scanning microscope (Olympus FLUOVIEW FV1000) coupled with an Olympus IX81 digital camera.

### 4.10 RNA‐seq analysis

The RNA‐seq experiments were performed by OE Biotech Co., Ltd. (Shanghai, China) using an Illumina HiseqTM 2500 system (Illumina, Inc., San Diego, CA, USA). Briefly, splenic MDSCs were isolated from ageing mice of the WT, *Tnfr1* ^−/−^, and *Tnfr2* ^−/−^ strains. A total of 1 × 10^6^ MDSCs were used for RNA extraction with the RNeasy Mini Kit (QIAGEN) and subjected to RNA‐seq analysis. The RNA samples were converted into a library of cDNA fragments, and Illumina sequencing adapters were added. Single‐end read sequences of 50 base pairs were acquired using the Illumina HiSeq system. The sequence reads underwent quality checks using FastQC. The data were analyzed using EdgeR software. Significantly differentially expressed genes were identified by setting padj < 0.05 and fold change >1.5.

### 4.11 Statistical analysis

Statistical significance was calculated using Student's *t*‐tests for comparisons between two groups, and one‐way ANOVA for comparisons involving more than two groups. *p* < 0.05 was considered to indicate a statistically significant difference. The Pearson correlation coefficient was used to investigate the linear relationship between two variables. Experiments were conducted without any exclusion criteria. To prevent selection bias, cell samples and genotype‐matched pools were randomly allocated into experimental groups. Data were analyzed automatically wherever feasible to eliminate subjective evaluations.

## AUTHOR CONTRIBUTIONS

Zhaoqing Wang and Zhihai Qin contributed to the study design, data processing, and manuscript writing. Ming Wang, Xiaohan Yao, Xixi Duan, Jiajia Wan, and Xiaohan Lou contributed to the conduction of biochemical and cellular experiments. Yijie Han, Peiguo Zheng, Yan Yan, Zhenzhen Pan, and Linyu Zhu contributed to the construction of mice model. Chen Ni, Zihao Wang, Lin Chen, and Fazhan Wang contributed to the statistical analysis. All authors read and approved the final version of the manuscript.

## CONFLICT OF INTEREST STATEMENT

The authors declare no conflict of interests.

## ETHICS STATEMENT

The study was conducted in accordance with informed consent guidelines from the patients and ethical standards set by the Ethics Committee of First Affiliated Hospital of Zhengzhou University (2020‐KY‐249).

## Supporting information

Supporting information

## Data Availability

The RNA‐seq data were uploaded in the Gene Expression Omnibus (GEO) database with the accession code GSE234791. All other data supporting the findings of this paper are available within the article and its Supplementary Information file.
