# Supplementary material for: Hyperexpression of tumor necrosis factor receptor 2 inhibits differentiation of myeloid‐derived suppressor cells by instigating apolarity during ageing
Source: MedComm (2020). 2024 Jun 12;5(6):e605. doi: 10.1002/mco2.605 (PMC11167233; doi:10.1002/mco2.605)
Supplement: Supplementary file 1 — Supporting information [file MCO2-5-e605-s001.docx]

# Supplementary Information

# Hyperexpression of tumor necrosis factor receptor 2 inhibits differentiation of myeloid-derived suppressor cells by instigating apolarity during ageing

Ming Wang^1^, Yijie Han^2^, Xiaohan Yao^1^, Xixi Duan^1^, Jiajia Wan^1^, Xiaohan Lou^1^, Yan Yan^1^, Peiguo Zheng^3^, Fazhan Wang^1^, Linyu Zhu^1^, Chen Ni^1^, Zhenzhen Pan^1^, Zihao Wang^1^, Lin Chen^1^, Zhaoqing Wang^*, 2^, Zhihai Qin^*, 1, 2^

^1^ Medical Research Center, The First Affiliated Hospital of Zhengzhou University, Zhengzhou University, Zhengzhou, 450052, Henan, China.

^2^ Key Laboratory of Protein and Peptide Pharmaceuticals, Institute of Biophysics, Chinese Academy of Sciences, Beijing 100101, China.

^3^ Clinical Laboratory, the First Affiliated Hospital of Zhengzhou University, Zhengzhou, Henan 450052, China

Correspondence: Z.H. Q. (Postal address: No. 1, Jianshe East Road, Zhengzhou, Henan, 450052, China and email: zhihai@ibp.ac.cn); Z.Q. W. (Postal address: No.15 Datun Road, Beijing 100101, China and email: wangzq@ibp.ac.cn).

# Supplementary materials

**Antibodies.** Anti-TNFR1 (Cat#13377), anti-TNFR2 (Cat# 3727), anti-JNK (Cat#9258s), anti-p-STAT3 (Cat#9145), anti-STAT3 (Cat# 9139), anti-GAPDH (Cat # 5174), anti-β-Actin (Cat # 4970), rabbit Anti-Mouse IgG (Cat # 58802), mouse Anti-rabbit IgG (Cat # 45262) antibodies were from Cell Signaling Technology. Anti-CD16/32 (Cat # 101302), FITC anti-mouse Gr1 (Cat # 4108405), PE anti-mouse TNFR2 (Cat# 358404), APC-R700 anti-mouse CD45 (Cat# 103128), BV510 anti-mouse ly6G/C (Cat# 108457), APC-cy7 anti-mouse/human CD11b (Cat# 101225), APC anti-mouse Ly-6G (Cat # 127614), PE anti-mouse Ly6C (Cat # 108406), Percp 5.5 anti- mouse CD11c (Cat # 117328), APC anti-human CD68 (Cat# 333810), Percp 5.5 anti-human CD11c (Cat# 337233), PE anti mouse CD45 (Cat# 304008) antibodies were from Biolegend. P16 Cat # ab51243, P21 Cat # ab188224 antibodies were from Abcam. p-JNK (Cat# MA5-15228), Secondary Antibody Donkey anti-Mouse IgG (H+L) Alexa Fluor® 488 (Cat # A-21202) antibodies were from Thermo Fisher Scientific. BV605 anti- mouse F4/80 (Cat # 123133), FITC anti- mouse CD80 (Cat# 104705) antibodies were from BD Bioscience. Percp 5.5 anti- human HLA-DR (Cat# 45-9956-42), FITC anti- human CD33 (Cat# 11-0337-42), PE-cy7 anti- human CD86 (Cat# 25-0869-42) antibodies were from eBioscience. Anti-P27 (Cat # a16633) antibody was from Ablconal. Anti-SCRIB antibody was from Novus Biologicals.

**Reagent.** DAPI (Cat # AR1176) was from Boster. Ficoll-Hypaque (Cat# GE17-1440-02) was from Sigma-Aldrich. EasySepTM HLA Chimerism Whole Blood CD33 Positive Selection Kit (Cat# 17885) and EasySep™ Mouse MDSC isolation kit (Cat# 19867) were from STEM cell Technologies. RBC lysis buffer (Cat# R1010), RIPA buffer (Cat# P1006), and BCA Protein Assay Kit (Cat# PC0020) were from Solarbio. RNAiso Plus (Cat # 9109), TB Green Premix Ex Taq II (Cat # RR820A) and PrimeScript RT Master Mix (Cat# RR036A) were from Takara. RPMI-1640 culture medium (Cat # C11875500BT) and Penicillin/ streptomycin (Cat # 15140122) were from Gibco. PGRN (Cat # HY-P74617) and SP600125 Cat # HY-12041 were from MCE. Protease inhibitor cocktail (Cat#9258s) and phenylmethylsulfonyl fluoride (PMSF, Cat#ST506) were from Beyotime. Fetal bovine serum (FBS, Cat # P30-1902) was PAN Biotech.

**Supplementary figures and legends**


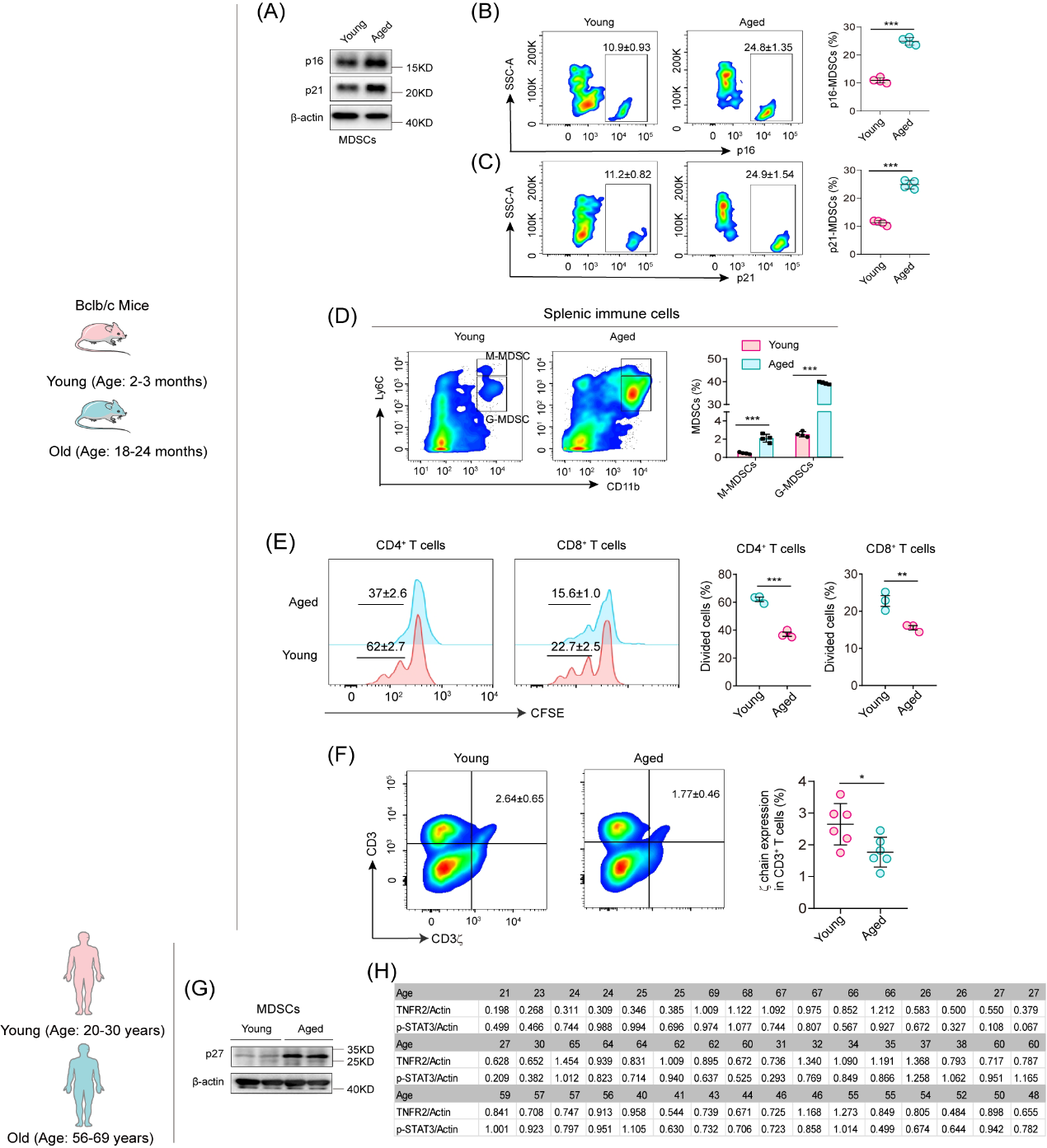


**Supplementary Figure 1. MDSCs senescence, accumulation and activation in old mice. (A)** Th**e** expression leves of p16 and p21 in MDSCs isolated from both young and old mice were determined by immunoblotting. (**B, C)** Flow cytometric analysis of p16 and p21 expression in MDSCs. Scatter plots showing individual percentage of p16 and p21 expression in MDSCs from both young and old mice (n=4). Data were expressed as mean ± SD and statistical analysis was performed using t-test. (**D**) Percentage of splenic M-MDSCs (Ly6C^hi^ CD11b^+^) and G-MDSCs (Ly6C^low^ CD11b^+^) in both young and old mice (n=4) was analyzed by flow cytometry. Data were expressed as mean ± SD and statistical analysis was performed using t-test. (**E**) MDSCs form young and old murine spleen (n=3) were co-cultured with CFSE-labeled splenic T cells for 3 days. Scatter plot showing individual percentage of proliferated CD4^+^ and CD8^+^ T cells measured by flow cytometry. Data were expressed as mean ± SD, with statistical analysis performed using t-test. (**F**) Flow cytometry analysis detected the percentage of splenic CD3^+^CD3ζ^+^ T cells in young and old mice (n=6). Data were expressed as mean ± SD and statistical analysis was performed using t-test. (**G**) Expression of p27 in human blood MDSCs from both young and old health donors was determined by immunoblotting. (H**)** Band densities of phosphorylated JNK and β-actin (in Fig, 6a) were measured and the ratio of p-JNK over β-actin was calculated. The data are from one representative experiment of more than three independent experiments (**B–F**). * *P* <0.05, ** *P* <0.01 and *** *P* <0.001.


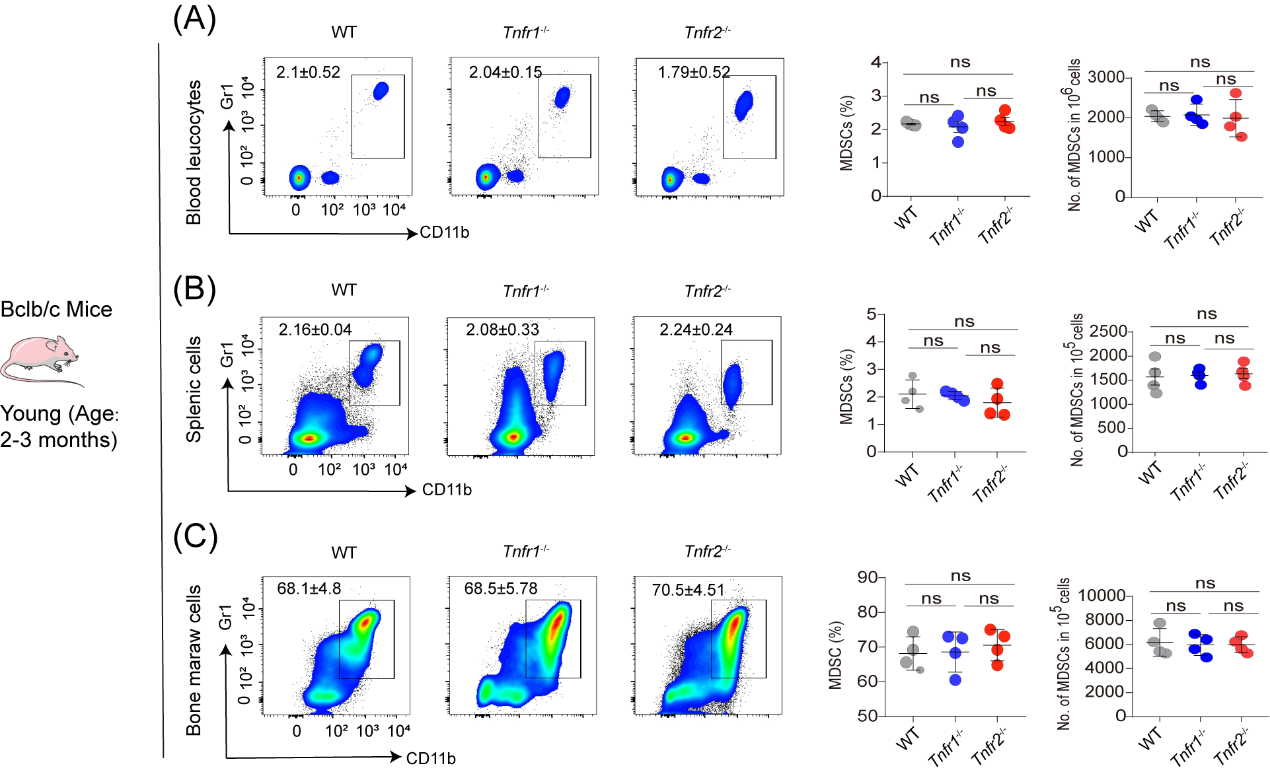


**Supplementary Figure 2. TNFR2 deficiency does not affect the proportion of MDSCs in young mice.** (**A-C**) Flow cytometric analyses were performed to detect the proportion of Gr1^+^CD11b^+^ MDSCs in blood (**A**), spleen (**B**) and bone marrow (**C**) from young wild-type, *Tnfr1*^-/-^ and *Tnfr2*^-/-^ mice (n=4). Scatter plot showing individual proportion. The data (mean ± SD) are from one representative experiment of more than three independent experiments (**A-C**). Statistical analysis was performed using one-way ANOVA, ns, not significant.


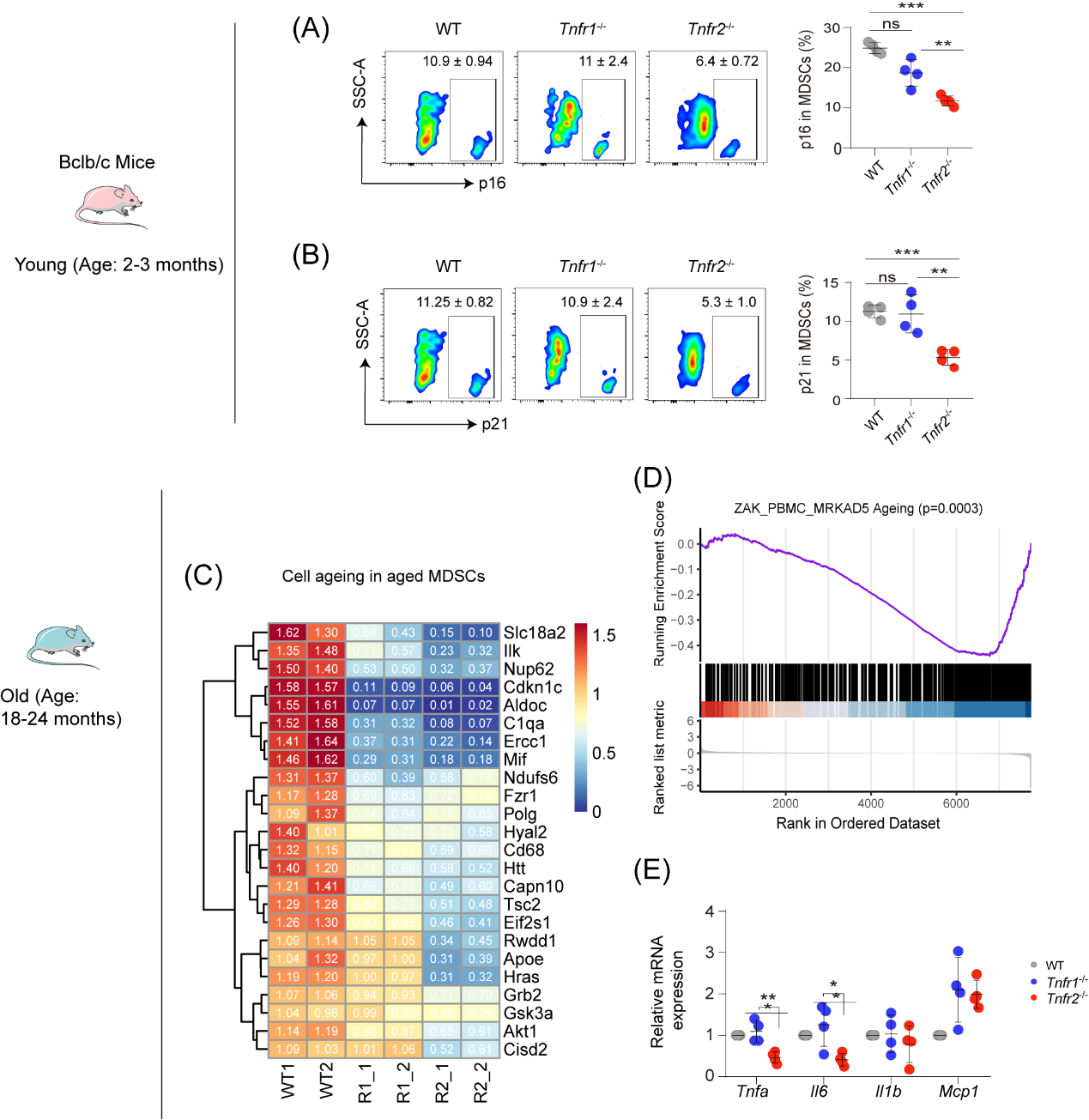


**Supplementary Figure 3. TNFR2 deficiency attenuates MDSCs’ ageing.** (**A, B**) Flow cytometry analysis of p16 and p21 expression in young splenic MDSCs isolated from wide type, *Tnfr1*^-/-^ and *Tnfr2*^-/-^ mice (n=4). Data are presented as mean ± SD and statistical analysis was performed using one-way ANOVA. (**C**) Heat map and hierarchical clustering of the ageing regulated genes in aged splenic MDSCs isolated from wide type, *Tnfr1*^-/-^ and *Tnfr2*^-/-^ mice. For each individual experiment, MDSCs were pooled from five mice, respectively. Data were obtained from two independent experiments. (**D**) Enrichment plots for ageing related data set enriched in GSEA wide type vs. *Tnfr2*^-/-^ group, reflecting the ranked list of the ageing regulated genes in the RNA-seq dataset. (**E**) Relative mRNA expression of *Tnfa, Il6, Il1b and Mcp1* in aged splenic MDSCs isolated from wide type, *Tnfr1*^-/-^ and *Tnfr2*^-/-^ mice (n=4) was measured using RT-PCR. The data are presented as mean ± SD and statistical analysis was performed using one-way ANOVA. The data are from one representative experiment of more than three independent experiments (**A, B**). * *P* <0.05, ** *P* <0.01 and *** *P* <0.001 and ns, not significant.


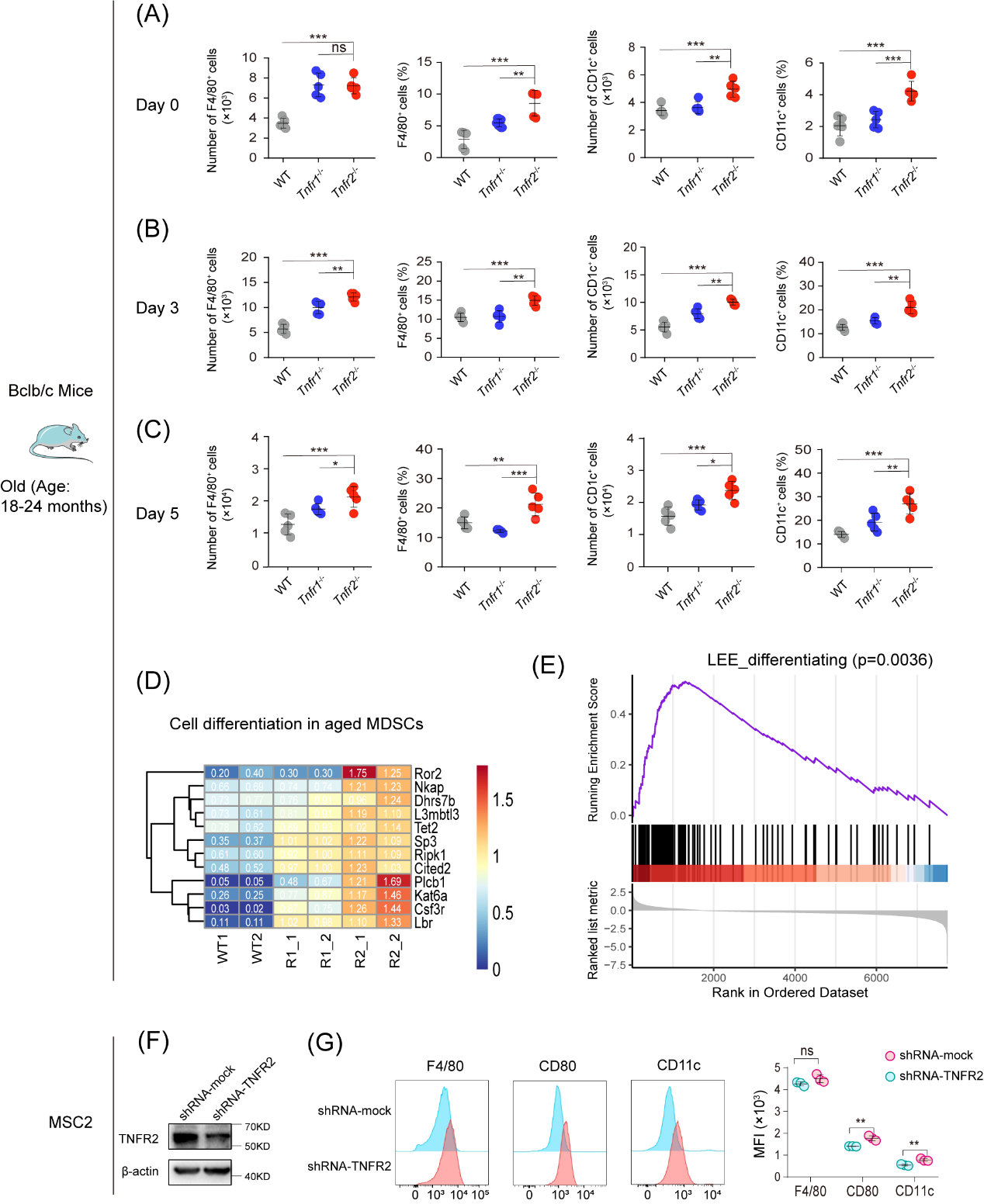


**Supplementary Figure 4. TNFR2 deficiency attenuates differentiation blockage of aged MDSCs.** (**A-C**) Old splenic cells were isolated from WT, *Tnfr1*^-/-^ and *Tnfr2*^-/-^ mice and further cultured for 3 or 5 days in the presence of GM-CSF (20 ng/mL**)**. The percentage of F4/80^+^CD1b^+^ macrophages and CD11b^+^CD11c^+^ dendritic cells in aged splenic cells at day 0, 3, and 5 were determined using flow cytometry analysis. (**D**) Heat map and hierarchical clustering of the cell differentiation regulated genes in aged splenic MDSCs. (**E**) Enrichment plots for cell differentiation related data set enriched in GSEA wide type vs. TNFR2^-/-^ group. (**F**) TNFR2 knockdown in MSC2 cells was validated by immunoblotting and equal loading was estimated by β-actin expression. (**G**) The expressions of F4/80, CD80, CD11c in MSC2 cells transfected with -shRNA-mock or shRNA-*Tnfr2* were detected by flow cytometry. Data are presented as mean ± SE and statistical analysis was performed using one-way ANOVA (**A-C**) or t-test (**G**). The data are from one representative experiment of more than three independent experiments (**A-C**). * *P* <0.05, ** *P* <0.01 and *** *P* <0.001 and ns, not significant.


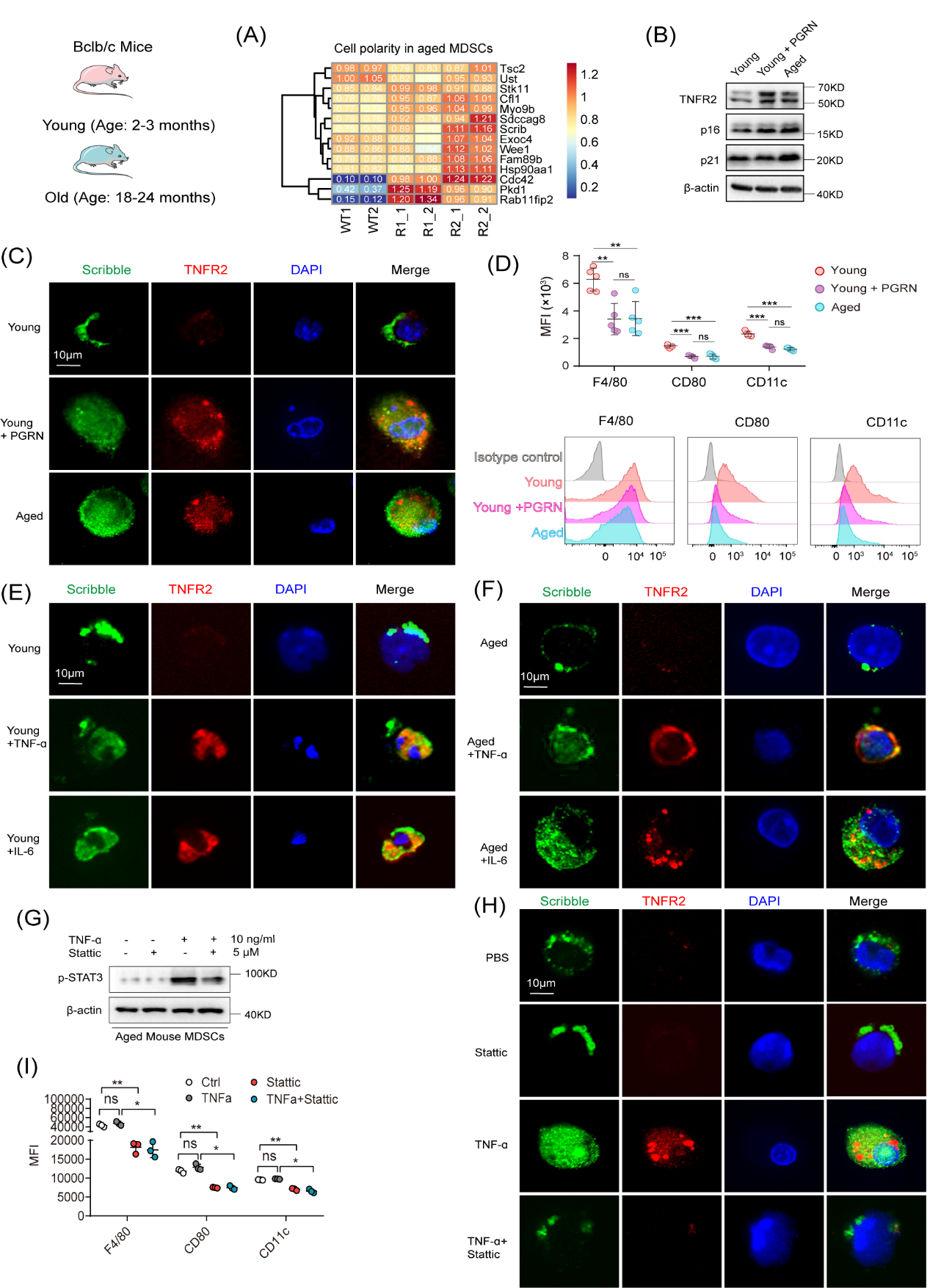


**Supplementary Figure 5. TNFR2 hyperexpression in young MDSCs impairs their proliferation, polarity, and differentiation.** (**A**) Enrichment plots for cell polarity related data set enriched in GSEA wide type vs. *Tnfr2*^-/-^ group. (**B-D**) MDSCs isolated from young and old murine spleens were treated with PGRN (200 ng/ml) for 24h. (**B**) Expression levels of TNFR2, p16 and p21 were determined by immunoblotting, equal loading was estimated by β-actin expression**.** (**C**) Localizations of Scribble (green) or TNFR2 (red) in MDSCs were analyzed by immunofluorescence staining. Nuclei was labeled with DAPI. Scale bar, 10 μm. (**D**) Flow cytometric analysis of the fluorescence intensity of F4/80, CD80, and CD11c in MDSCs. (E) Isolated MDSCs from mouse spleen were exposed to TNF-α (10 ng/ml) or IL-6 (5 ng/ml) for 1 hour. Localizations of Scribble (green) or TNFR2 (red) in MDSCs were analyzed by immunofluorescence staining. Nuclei was labeled with DAPI. Scale bar, 10 μm. (F-H) Stimulation of mouse splenic MDSCs with TNF-α (10 ng/ml), static (5 μM), or a combination. (G) Western blots depict the phosphorylation status of STAT3 after 1 hour of treatment. (F, H) Localizations of Scribble (green) or TNFR2 (red) in MDSCs were analyzed by immunofluorescence staining. Nuclei was labeled with DAPI. Scale bar, 10 μm. (I) Flow cytometric analysis of fluorescence intensity for F4/80, CD80, and CD11c in MDSCs after 24 hours. Data are from one representative experiment of more than three independent experiments, presented as mean ± SD, and statistically analyzed using one-way ANOVA. ** *P* <0.01, *** *P* <0.001, and ns, not significant.


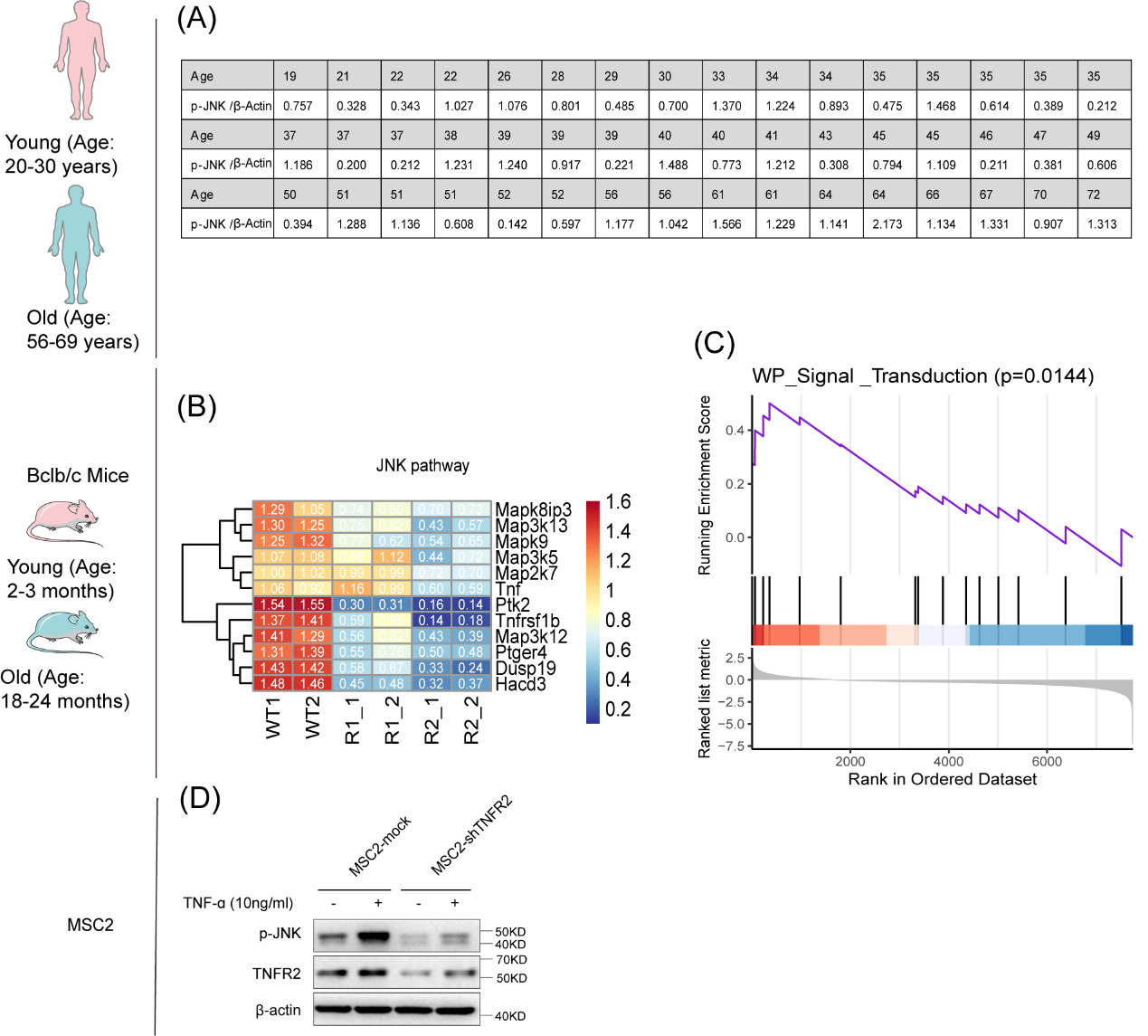


**Supplementary Figure 6. JNK activation is positively correlated with TNFR2 expression.** (**A**) Band densities of phosphorylated JNK and β-actin (in Figure 6A) were measured and the ratio of p-JNK over β-actin was calculated. (**B**) Heat map and hierarchical clustering of the JNK pathway regulated genes in aged splenic MDSCs. (**C**) GSEA was performed in the wide type vs. TNFR2^-/-^ groups. The GSEA algorithm calculates an enrichment score reflecting the degree of signal transduction ranked list of the genes in the RNA-seq dataset. (**D**) TNFR2 expression and JNK activation levels in MSC2 cells transfected with -shRNA-*Tnfr2* with or without TNF-α (10 ng/ml) treatment for 30 min were determined by immunoblotting, equal loading was estimated by β-actin expression.


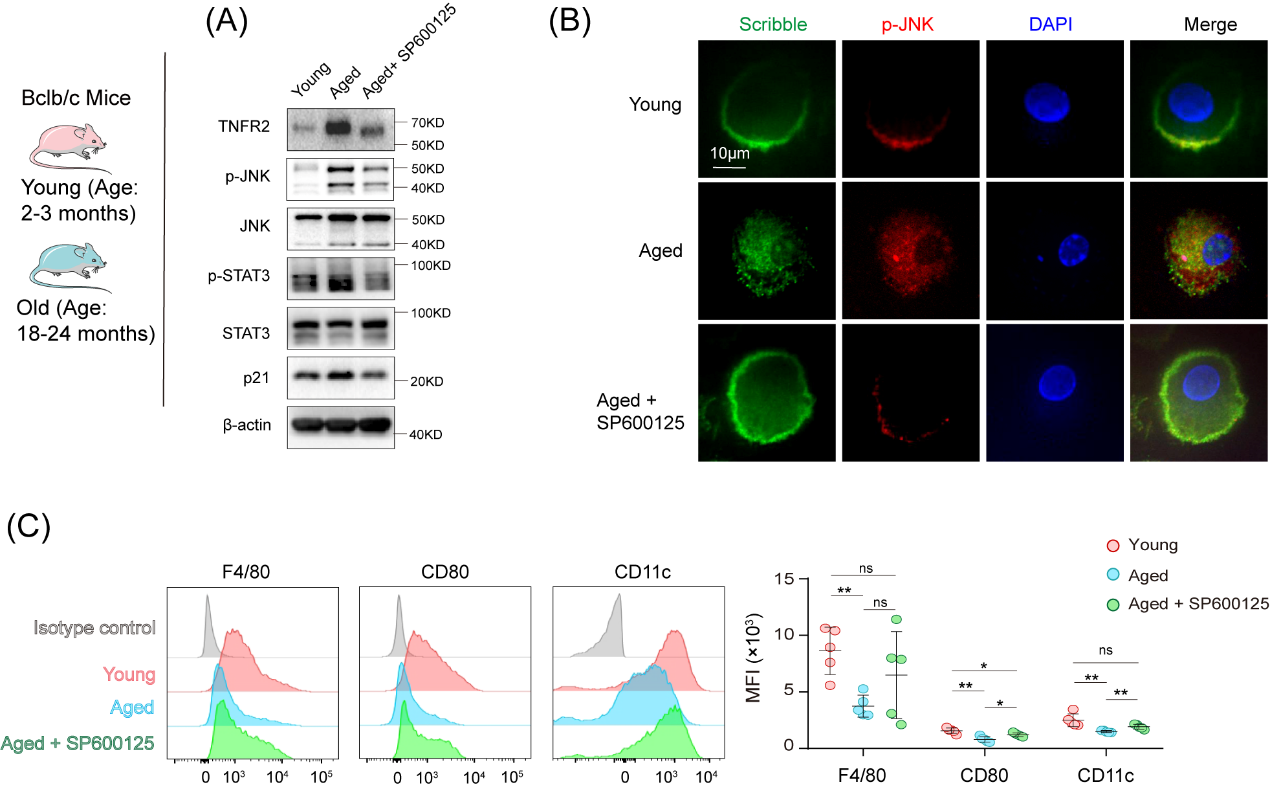


**Supplementary Figure 7. Inhibition of JNK in MDSCs restores their polarity and mature differentiation.** MDSCs were isolated from the spleen of young and old mice and aged MDSCs were treated with SP600125 (40 nM). (A) Abundance of TNFR2, p-JNK, JNK, p-STAT3, STAT3, p16 and p21 was determined by immunoblotting using respective antibodies and β-actin was used as equal loading control. (B) Immunostaining of Scribble and p-JNK in MDSCs and nuclei was labeled with DAPI. Scale bar, 10 μm. (**C**) Flow cytometric analysis of the fluorescence intensity of F4/80, CD80, and CD11c in MDSCs. The data are from one representative experiment of more than three independent experiments and are presented as mean±SE (n=5). Statistical analysis was performed using one-way ANOVA. * *P* <0.05, ** *P* <0.01, and ns, not significant.


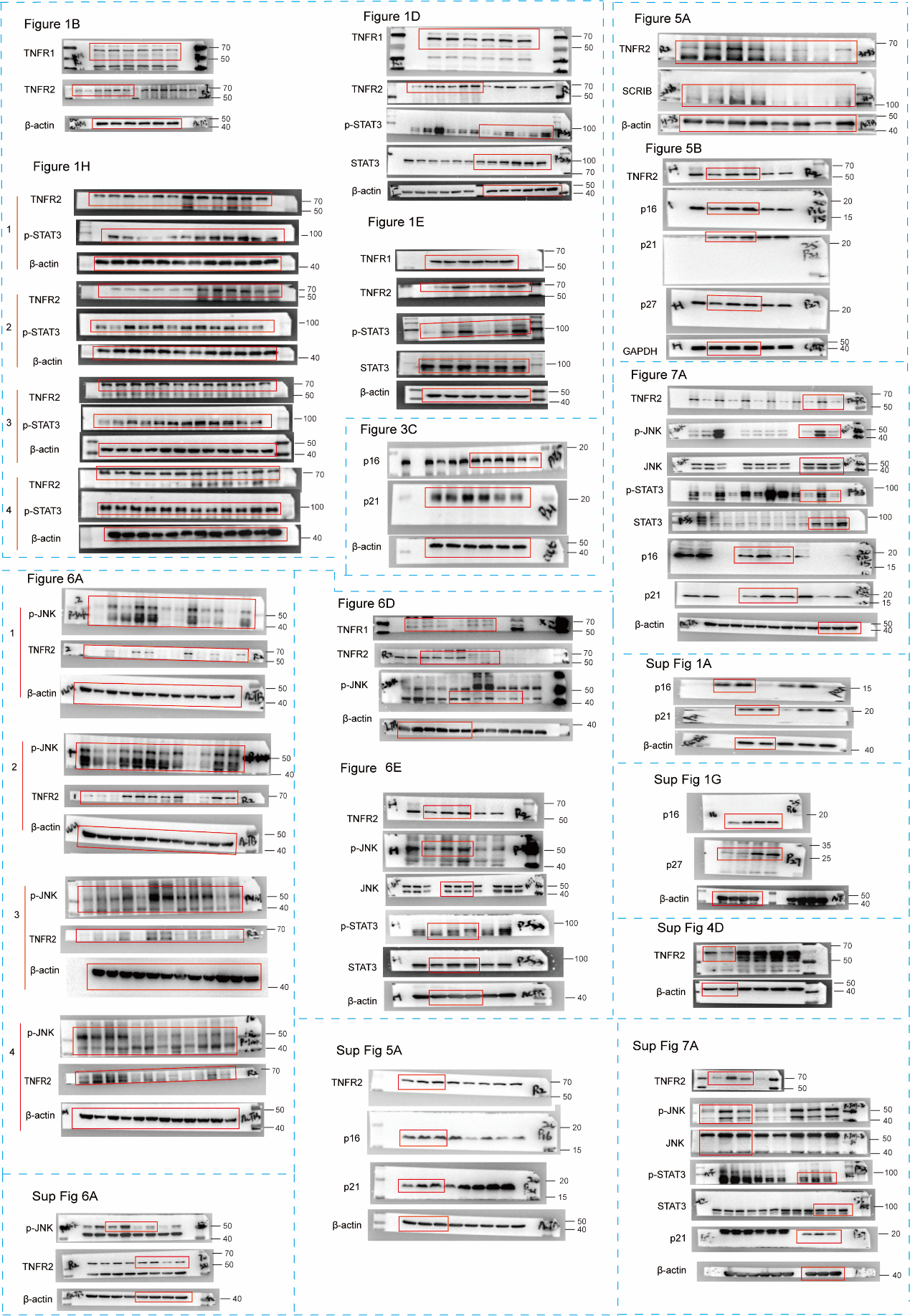


**Supplementary Figure 8.** All uncropped versions of the Western Blots are presented in the same order as in the corresponding figures.
